# Microalgae-based biofertilizers improve fertility and microbial community structures in the soil of potted tomato

**DOI:** 10.3389/fpls.2024.1461945

**Published:** 2024-12-23

**Authors:** Xiaotong Song, Jiayi Liu, Yanzhang Feng, Chengxu Zhou, Xiaohui Li, Xiaojun Yan, Roger Ruan, Pengfei Cheng

**Affiliations:** ^1^ College of Food Science and Engineering, Ningbo University, Ningbo, Zhejiang, China; ^2^ School of Marine Sciences, Ningbo University, Ningbo, Zhejiang, China; ^3^ Center for Biorefining, Department of Bioproducts and Biosystems Engineering, University of Minnesota-Twin Cities, Saint Paul, MN, United States

**Keywords:** *Tribonema* sp., microalgae-based biofertilizers, agroforestry waste, soil physicochemical characteristics, soil microbial functions

## Abstract

Continuous cropping decreases soil nutrients and destroys microbial community structure, so the development of eco-friendly and effective biofertilizers is necessary under present conditions. In this study, the preserving microalgal strain *Tribonema* sp. (H) was firstly selected to be combined with agroforestry waste (shell powder, straw fermentation liquid) and the agroforestry microorganism *Bacillus* sp. to form microalgae-based fertilizers for the continuous cropping soil of potted tomato. Compared to the control (CK), microalgae-based fertilizers (concentration: 4.45 × 10^6^ cells/ml, dosage: 20 ml/day) improved soil nutrients and salinization indicators. Specifically, the combination of *Tribonema* sp. and shell powder (HB) reduced electrical conductivity (EC) by 33.7% and significantly increased the Ca^2+^ content by 59.4%; *Tribonema* sp. and *Bacillus* sp. (HY) improved the effects of available phosphorous (AP), DOC, DON, NH_4_^+^-N, NO_3_^−^-N, and Mg^2+^ in the soil by 27.4%, 231.3%, 403.4%, 125.2%, 215.6%, and 73.4%, respectively. Microalgae-based fertilizers alter the abundance of soil bacteria and fungi, causing beneficial bacteria such as *Thermonaerobaculia*, *Subgroup_10*, *Sordariomycetes*, and *Microascaceae* to increase, while pathogenic bacteria like *Pseudomonas*, *Togniniaceae*, and *Phaeoacremonium* decreased. Combining microalgae with agroforestry wastes as a biofertilizer is promising to improve the microbial community structure of the soil with continuous cropping, which will aid in the increase of tomato production and promote green agricultural development.

## 1 Introduction

Tomatoes (*Lycopersicon esculentum*) are nutritious, flavorful, and versatile, suitable for both raw consumption and cooking. Tomatoes are rich in vitamins, minerals, and other essential elements, promote digestion and detoxification, nourish the blood, and enhance the appetite; they can also be processed into various products such as sauces and canned goods (Coelho et al., 2023). China is the largest tomato-producing country in the world, and the widespread use of “smart farming” in China makes tomato cultivation more controlled (Ya-Dan et al., 2017). Potted tomato plants are visually appealing with vibrant colors, making them widely popular and valuable for ornamental purposes. They can be grown in greenhouses, balconies, conservatories, terraces, and well-lit indoor spaces. However, potted tomatoes face challenges such as limited suitable varieties, poor product quality, and low production technology (Xin et al., 2019). For traditional management systems, continuous monoculture of tomatoes in greenhouses has negative impacts on soil quality, leading to stunted plant growth, poor fruit quality, decreased yield, and increased pest infestations (Panno et al., 2021). Studies have shown that continuous cropping can deplete soil nitrogen, resulting in reduced grain yields and protein content (Dalal et al., 2018), as well as a significant decrease in soil organic carbon, total nitrogen, potential mineralizable nitrogen, and microbial content (Norkaew et al., 2019). Continuous cultivation can increase soil phenolic compounds, lower pH, exacerbate soil acidification (Sahab et al., 2021), and consequently promote soil-borne pathogenic fungi growth and decrease beneficial microbes (He et al., 2021). Gao et al. found that continuous cultivation significantly reduces soil urease and sucrase activities, enhancing the complexity of the microbial network (Gao and Zhang, 2023). Therefore, it is essential to use efficient green organic fertilizers to improve soil fertility, crop resistance, microbial activity, and other factors in continuous cropping systems.

Agricultural and forestry waste, as a renewable resource, can serve as excellent organic biofertilizers, effectively improving soil quality and fertility (Anastopoulos et al., 2019). Processing of discarded shells from aquaculture into shell powder can enhance soil pH, increase bacterial and fungal diversity, and improve crop growth in acidic soils (Zhao et al., 2022). Field straw, through carbon sequestration, increases soil inorganic and organic carbon content, as well as soil conductivity and pH (Battaglia et al., 2022). Correspondingly, fermented straw liquid can significantly boost potted plant growth, soil properties, and catalase (CAT) enzyme activity (Zhou et al., 2020). Deep plowing and returning straw to the field could effectively enhance soil phospholipid fatty acids (PLFAs) of bacteria, actinomycetes, and fungi, along with the activities of urease, invertase, and polyphenol oxidase, leading to increased grain yield (Ning et al., 2021). Microbial agents such as *Bacillus subtilis* can improve soil fertility, reduce soil hardening, and increase microbial diversity. It was found that using a microbial-induced calcium carbonate precipitation (MICP) alongside *B. subtilis* treatment effectively reduces soil erosion due to wind and improves soil fertility, without any adverse effects on soil and crops (Namdar-Khojasteh et al., 2022). Therefore, utilizing agricultural and forestry waste to improve soil quality can promote resource recycling effectively.

Microalgae can synthesize various active substances through photosynthesis, promote soil phosphorus cycling through nitrogen fixation, and thereby affect soil microorganisms to achieve the effects of promoting plant growth, biological control, and soil stabilization (Bibi et al., 2024). They are potential regulators of continuous cropping obstacles (Alvarez et al., 2021; Zhang et al., 2018; Sathinathan et al., 2022). In addition to improving soil structure and retaining soil moisture and other physical properties, microalgae can also be used as organic fertilizers (microbial inoculants) and applied to soil, seeds, or plant surfaces. Through the activities of live cells, they can enhance the supply or availability of plant nutrients, thereby improving plant growth (Balasubramaniam et al., 2021). Nayak et al. found that the use of de-oiled microalgal biomass as a biofertilizer in potted rice effectively increased plant height, branch number, and rice yield compared to the application of chemical fertilizer alone, thus reducing the amount of chemical fertilizer used (Nayak et al., 2019). However, the cost of microalgae as a biological fertilizer needs to be considered. It is worth trying to explore the resource utilization of microalgae coupled with other wastes as a biofertilizer.

Building on these findings, this paper first attempts to combine microalgae with agricultural and forestry waste (shell powder, straw fermentation liquid) and the agroforestry microorganism *Bacillus* sp. to form an algae-based biofertilizer. It examines the effects of algae-based biofertilizer on the physicochemical properties and microbial community structure in the soil of potted tomato. The aim is to find a green and efficient organic fertilizer that can be used complementary to fertilizer production and to improve the utilization of agricultural and forestry waste resources, thereby laying a theoretical foundation and providing practical guidance for agricultural production applications.

## 2 Materials and methods

### 2.1 Screening of microalgae-based biofertilizers

The microalgae strain *Tribonema* sp. used in this experiment was preserved and provided by Ningbo University, shell powder was provided by Xinlei Mineral Powder Processing Company, straw fermentation broth was provided by Yunjiao Biotechnology Co., Ltd., and *Bacillus* sp. was preserved and provided by Ningbo University. *Tribonema* sp. was cultured at 25°C ± 2°C, with a light intensity of 3,000 ± 50 lux and a light–dark cycle of 12 h:12 h (Cheng et al., 2020). Bacterial medium was prepared and poured into plates for cooling and standby use. The preserved strain *Bacillus* sp. was activated by the three-zone streaking method, followed by scraping individual colonies on a sterile bench and transferring them to Luria–Bertani (LB) broth. The bacteria were then incubated in an incubator at 37°C for 12 h for subsequent experiments. *Tribonema* sp. (H) was cultured with shell powder (B) at different ratios (0, 2%, 4%, 6%, 8%) (HB), with straw fermentation broth at different ratios (0, 2%, 5%, 10%, 15%) (HJ), and with *Bacillus* sp. at different ratios (0, 3:1, 6:1, 9:1, 12:1, 15:1) (HY) during the logarithmic growth phase of the microalgae. The cell count and chlorophyll content were measured to screen for the best microalgae-based biofertilizer.

### 2.2 Irrigation of tomato soil with microalgae-based biofertilizer

Soil for the potting experiments was collected from a sample of an experimental field in Zhejiang Academy of Agricultural Sciences (29°46′228″N, 121°39′198″E) that had been cropped for 7 years. This region has a subtropical climate with an average annual temperature of 18.7°C and an average annual precipitation of 1,653.74 mm. The basic parameters of the upper soil layer (0–20 cm) in the field at the baseline level are shown in **Table 1**. The fresh soil was dried and screened through a 10-mesh sieve, and 200~300 g was weighed into culture bottles. Three biological replicates were set up for each group. Distilled water was added to the control group (CK), while *Tribonema* sp. was added to HB. The HB treatment consisted of 4% proportion of shell powder added to the logarithmic *Tribonema* sp., the HJ treatment consisted of 5% proportion of straw fermentation broth added during the logarithmic phase of *Tribonema* sp., and the HY treatment consisted of an optimal ratio of *Tribonema* sp. to *Bacillus* sp. of 12:1. Each of the three 20 ml microalgae-based biofertilizers was used daily to irrigate the soil.

**Table 1:** Initial physical and chemical properties in the soil of potted tomato with continuous cropping.

	TC (g kg^−1^)	TN (g kg^−1^)	AP (mg kg^−1^)	DOC (g kg^−1^)	DON (g kg^−1^)	NH_4_^+^-N (mg kg^−1^)	NO_3_^−^-N (mg kg^−1^)
CK	28.25 ± 0.84	2.76 ± 0.11	65.6 ± 3.57	51.80 ± 11.4	73.46 ± 3.91	1.25 ± 0.8	68.04 ± 5.81
	pH (mol l^−1^)	EC (ms cm^−1^)	K^+^ (g kg^−1^)		Na^+^ (g kg^−1^)	Ca^2+^ (g kg^−1^)	Mg^2+^ (mg kg^−1^)
CK	6.84 ± 0.05	1.42 ± 0.38	0.25 ± 0.03		0.03 ± 0.02	4.67 ± 0.13	0.43 ± 0.06

### 2.3 Analysis of the biochemical composition of microalgae

Microalgae were cultured until the logarithmic growth phase was completed. Then, the agricultural and forestry waste (shell powder, straw fermentation products) and *Bacillus* sp. were added. The biomass and chlorophyll content of the algae cells were determined by the improved method, and the microalgae–bacteria co-culture fermentation broth was measured according to the method of Lakatos et al (Cheng et al., 2020; Lakatos et al., 2017).

### 2.4 Measurement of soil indices

Liquid microalgal cells from microalgae-based biofertilizer were used to irrigate the soil of the tomato plants. After continuous irrigation for 60 days, the physicochemical indices and microbial community structure in the soil of potted tomato were measured.

#### 2.4.1 Measurement of soil physical and chemical properties

Ten grams of fresh soil was collected to determine the contents of DOC, DON, NH_4_^+^-N, and NO_3_^−^-N. Air-dried soil was used to measure pH, electrical conductivity (EC), total carbon (TC), total nitrogen (TN), available phosphorous (AP), and the soluble cations K^+^, Na^+^, Ca^2+^, and Mg^2+^. All soil chemical property measurement methods were consistent with those reported by Chen et al. (2022).

#### 2.4.2 DNA extraction and amplification fragment sequencing

Rhizosphere soil (0.5 g) was used for DNA extraction using the PowerSoil DNA Isolation Kit (MO BIO, San Diego, USA). The concentration of the extracted DNA was determined using a Thermo NanoDrop One spectrophotometer (Thermo Fisher Scientific, Wilmington, USA). The DNA was stored at −20°C. Using genomic DNA as a template, PCR amplification was performed with barcoded specific primers and TaKaRa Premix Taq^®^ Version 2.0 (TaKaRa Biotechnology Co., Dalian, China), depending on the selected sequencing region. The primer target regions were 16S V4 region primers (515F and 806R), 18S V4 region primers (528F and 706R), and ITS1 primers (ITS5-1737F and ITS2-2043R). The fragment length and concentration of the PCR products were detected using 1% agarose gel electrophoresis. The concentrations of the PCR products were compared using GeneTools Analysis Software (Version 4.03.05.0, SynGene), and the volumes required for each sample were calculated based on equal mass principles. The PCR products were then mixed together. The PCR mixture was recovered using the E.Z.N.A.^®^ Gel Extraction Kit (Omega, USA) with TE buffer elution to recover the target DNA fragments. Library preparation was performed following the standard protocol of the NEBNext^®^ Ultra™ II DNA Library Prep Kit for Illumina^®^ (New England Biolabs, USA). The constructed amplicon libraries were sequenced using PE250 on the Illumina Nova 6000 platform (Guangdong Magigene Biotechnology Co., Ltd., Guangzhou, China).

#### 2.4.3 Sequencing process

All valid tags that were replicated three times in all the samples were clustered using uparse (Edgar, 2013). Sequences were quality bleached and denoised, and chimeras were removed using DADA2 (Callahan et al., 2016) provided in the QILLME2 (Version 2020.11.0) process. For species annotation of representative sequences, each OTU’s representative sequence was aligned with the SILVA (16S), RDP (16S), Greengenes (16S), SILVA (18S), and Unite (ITS) databases using Usearch-sintax, with a default confidence threshold of 0.8. The taxonomy results of species annotation were classified into seven levels: kingdom, phylum, class, order, family, genus, and species. The phylogenetic relationships of all OTU representative sequences were rapidly aligned using MUSCLE (Edgar, 2004) (Version 3.8.31). Finally, the data for each sample were homogenized based on the minimum amount of data in any sample. Alpha diversity and beta diversity analyses were then performed.

### 2.5 Data analysis

The experimental materials were entrusted to Guangdong Meige Genomics Technology Co., Ltd. for high-throughput sequencing. We used R software (V5.1.3) to calculate the union number (Pan) and intersection number (Core) of the target classification level in different sample sizes to assess whether the sample size was sufficient. Based on the OTU abundance table, we used usearch-alpha_div (V10, http://www.drive5.com/usearch/) to represent the diversity of the microbial community through three diversity indices (richness, Shannon_2, Chao1) (Wu et al., 2021). We conducted intergroup difference analysis of alpha diversity indices using R software, selected the Kruskal–Wallis rank sum test, and performed principal coordinate analysis (PCoA) to determine the beta diversity of the tomato soil microbial community. Significant differences were tested using analysis of similarities (ANOSIM) (Sun et al., 2021). The significance of the correlation between other microbial communities and physicochemical variables was tested using Spearman’s test. STAMP v2.1.3 was used to compare the microbial composition in tomato soil with and without fertilization. We used LEfSe (http://huttenhower.sph.harvard.edu/galaxy) to identify bacterial and fungal biomarker genera in the soil with and without fertilization. Finally, we used the FUNGuild tool to predict the functional diversity of fungal communities in different groups. All tests in this study were repeated three times.

## 3 Results

### 3.1 Preparation of microalgae-based biofertilizers

The logarithmic stage of *Tribonema* sp. was cultured with shell powder, straw fermentation broth, and *Bacillus* sp. at different ratios (as described in Section 2.1). The same initial density was set for each group, and the cell density and chlorophyll content of microalgae were measured every 2 days.

**Figure 1:**
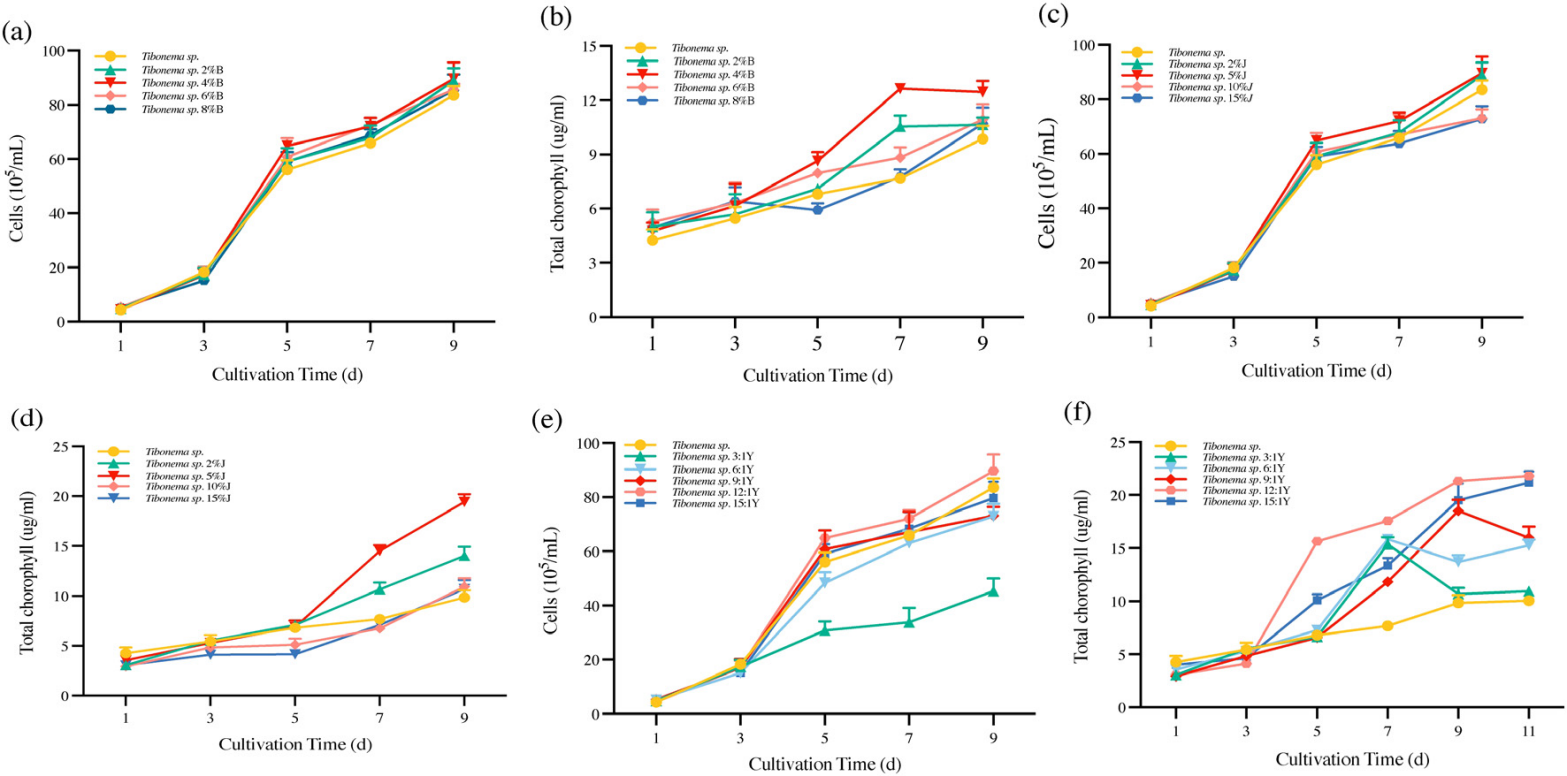
Preparation of the microalgae-based biofertilizer. **(A, B)** *Tribonema* sp. with shell powder; **(C, D)** *Tribonema* sp. with straw fermentation liquid; **(E, F)** *Tribonema* sp. with *Bacillus* sp.

When 4% shell powder was added, the biomass and chlorophyll content of *Tribonema* sp. reached 4.45 × 10^6^ cells/ml and 13.11 µg/ml, respectively, showing significantly better growth compared to the other tested conditions (**Figures 1A, B**). When 5% of straw fermentation broth was added to the *Tribonema* sp., the biomass and chlorophyll content were 4.47 × 10^6^ cells/ml and 18.21 µg/ml, respectively (**Figures 1C, D**), which were significantly better than the other proportions of fermentation broth.

*Tribonema* sp. was co-fermented with *Bacillus* sp., reaching an OD value of approximately 0.6 after 12 h of incubation. The microalgae were first cultivated to the logarithmic phase and then co-cultured with *Bacillus* sp. at various microalgae-to-bacteria ratios (0, 3:1, 6:1, 9:1, 12:1, and 15:1) to assess biomass and chlorophyll content. Among the tested ratios, the 12:1 ratio exhibited the most favorable growth conditions, with a biomass of 4.45 × 10^6^ cells/ml by day 9 and a chlorophyll content of 21.59 µg/ml (**Figures 1E, F**).

### 3.2 Effects of microalgae-based biofertilizers on soil nutrients

The screened microalgae-based biofertilizers were applied to tomato soil that had been continuously cropped for 7 years, and the soil nutrients were measured. As shown in **Table 2**, the microalgae-based biofertilizers were able to increase the content of TC and TN in the soil. In particular, the microalgae-based biofertilizers significantly enhanced the content of AP in the soil. When co-cultivated with agricultural and forestry waste, the HB combination demonstrated superior performance compared to the other groups, increasing the AP content by 27.4%. Additionally, **Table 2** shows that the addition of microalgae-based fertilizers significantly altered the content of DOC, DON, NH_4_^+^-N, and NO_3_^−^-N in the soil. Among them, the microalgae-based fertilizer HB had the most significant effects, increasing these by 231.3%, 403.4%, 125.2%, and 215.6%, respectively, and outperforming the addition of *Tribonema* sp. alone. This suggests that algae-based biofertilizers can significantly increase the content of nutrients in the soil.

**Table 2:** Comparison of inter-root soil nutrients for potted tomato with 60-day drip irrigation.

	TC (g kg^−1^)	TN (g kg^−1^)	AP (mg kg^−1^)	DOC (g kg^−1^)	DON (g kg^−1^)	NH_4_^+^-N (mg kg^−1^)	NO_3_^−^-N (mg kg^−1^)
CK	25.24 ± 1.06	2.77 ± 0.09	58.2 ± 4.19	40.5 ± 15.2	43.1 ± 5.21	1.07 ± 0.76	58.1 ± 7.31
H	25.84 ± 1.52	3.25 ± 0.71	68.9 ± 1.66	73.4 ± 5.13	128.6 ± 9.37	1.86 ± 0.64	129.4 ± 17.9
HB	27.43 ± 1.60	3.19 ± 0.82	70.8 ± 0.47	88.9 ± 7.2	168.3 ± 7.31	1.79 ± 0.18	173.0 ± 18.4
HJ	28.92 ± 0.65	3.31 ± 0.17	71.0 ± 1.05	106.9 ± 11.7	189.6 ± 9.08	2.01 ± 0.78	162.5 ± 13.5
HY	27.91 ± 0.48	3.24 ± 1.72	74.2 ± 0.96	134.2 ± 15.9	217.3 ± 9.08	2.41 ± 1.76	183.4 ± 21.3

Water: CK; H: *Tribonema* sp.; HB: *Tribonema* sp. and shell powder; HJ: *Tribonema* sp. and straw fermentation liquid; HY: *Tribonema* sp. and *Bacillus* sp.

### 3.3 Effects of microalgae-based biofertilizers on soil salinization

Tomato plants were irrigated with selected microalgae-based biofertilizers, and the soil salinization indices were measured (**Table 3**). After the application of microalgal fertilizers, the soil maintained a neutral pH. The HB powder treatment significantly reduced electrical conductivity by 33.7% and increased the Ca²^+^ content in the soil by 59.4%. Additionally, the HY combination significantly increased the Mg²^+^ content by 73.4%.

**Table 3:** Comparison of salinity properties of inter-root soils for potted tomato with 60-day drip irrigation.

	pH (mol l^−1^)	EC (ms cm^−1^)	K^+^ (g kg^−1^)	Na^+^ (g kg^−1^)	Ca^2+^ (g kg^−1^)	Mg^2+^ (mg kg^−1^)
CK	7.23 ± 0.07	1.54 ± 0.43	0.25 ± 0.04	0.08 ± 0.02	4.18 ± 0.07	0.51 ± 0.08
H	7.19 ± 0.06	1.21 ± 0.13	0.52 ± 0.11	0.28 ± 0.04	8.31 ± 0.25	1.23 ± 0.09
HB	7.43 ± 0.27	1.02 ± 0.04	0.57 ± 0.08	0.24 ± 0.02	10.32 ± 0.28	1.07 ± 0.13
HJ	7.13 ± 0.18	1.13 ± 0.07	0.58 ± 0.03	0.23 ± 0.06	8.56 ± 0.04	1.31 ± 0.09
HY	7.27 ± 0.09	1.54 ± 0.25	0.61 ± 0.12	0.33 ± 0.05	9.17 ± 0.13	1.92 ± 0.15

Water: CK; H: *Tribonema* sp.; HB: *Tribonema* sp. and shell powder; HJ: *Tribonema* sp. and straw fermentation liquid; HY: *Tribonema* sp. and *Bacillus* sp.

### 3.4 Diversity and composition of microbial communities

**Figure 2:**
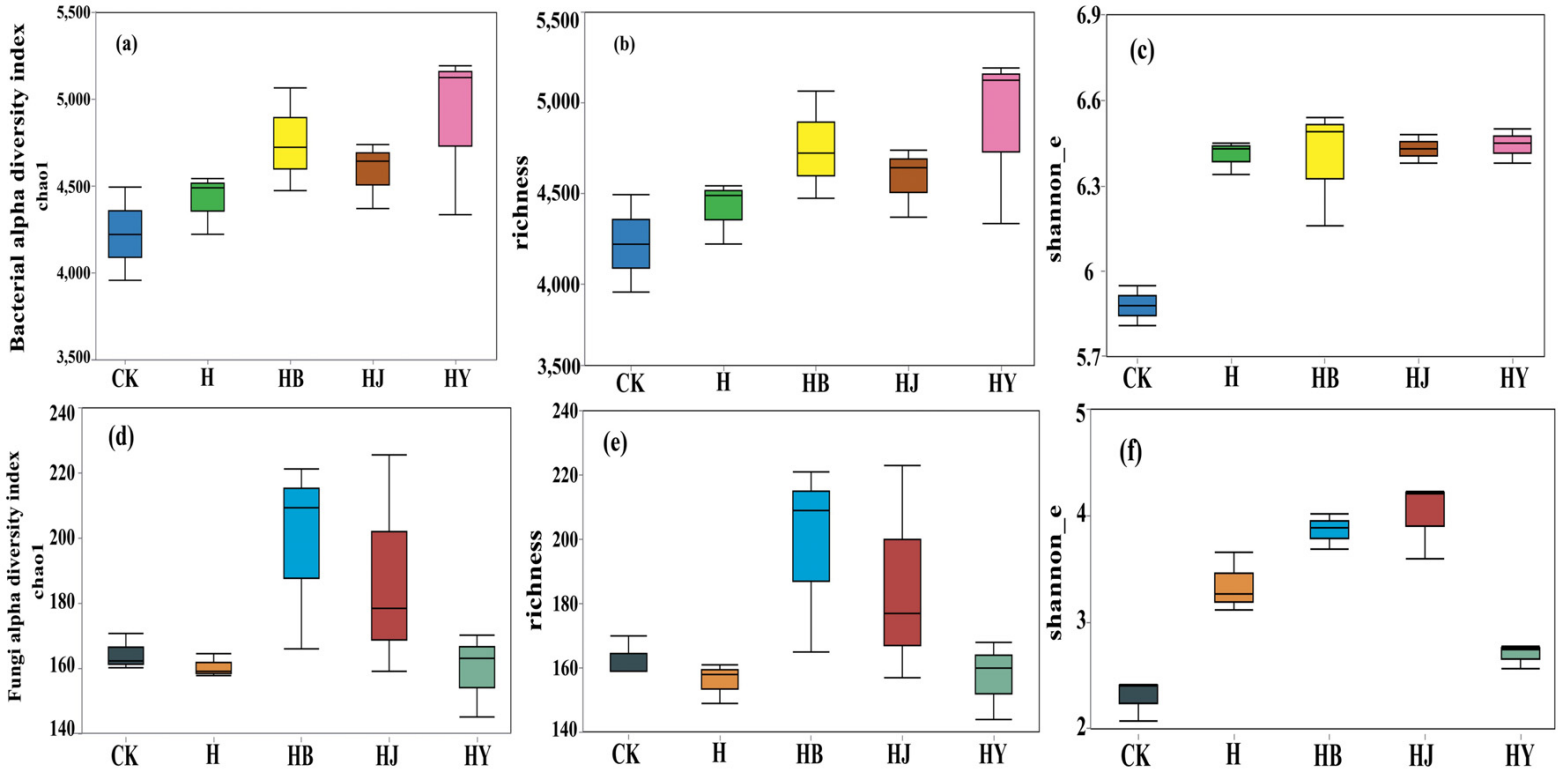
Effects of microalgae-based fertilizers on bacterial and fungal community diversity in the soil of potted tomato. **(A, D)** Chao1 index; **(B, E)** richness index; **(C, F)** Shannon index.

After sequencing, all samples were normalized based on the lowest number of reads, and downstream analysis was conducted using the normalized data. The bacterial richness following the HB, HJ, and HY treatments was significantly higher than that observed in the CK treatment, with the HY treatment showing the highest bacterial richness (**Figure 2**). This suggests that algae-based biofertilizers substantially enhance the richness and diversity of bacterial communities in tomato soil. In contrast, there were no significant differences in the richness and diversity of tomato endophytic fungi across the various microalgal treatments (**Figure 2**).

**Figure 3:**
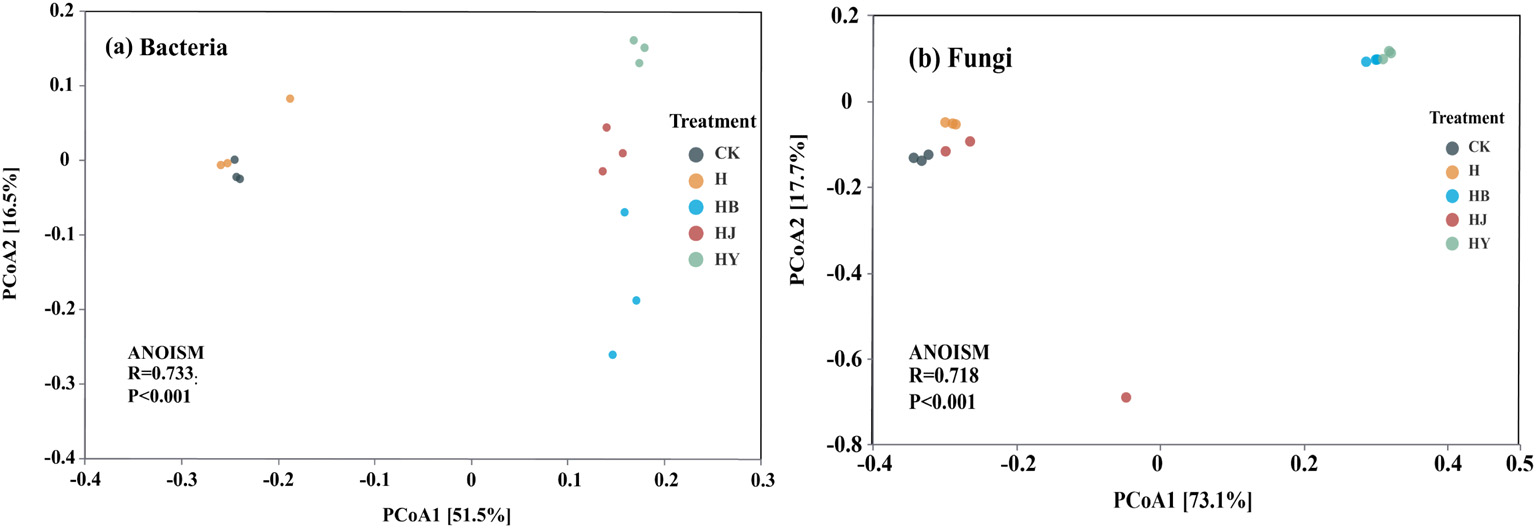
Structural changes in bacterial and fungal communities in the soil of potted tomato treated with different microalgae-based fertilizers [principal coordinate analysis (PCoA) based on the Bray–Curtis difference matrix]. **(A)** Bacteria PCoA analysis; **(B)** fungi PCoA analysis.

The CK and H treatments clustered closely together, while the other treatments were more dispersed (**Figure 3A**). These results indicate significant differences in the bacterial community structure of tomato soil across the various microalgal treatments. For fungal communities, CK, H, and HJ treatments formed one cluster, whereas HB and HY treatments clustered separately, highlighting notable differences in fungal community structure among the different microalgal treatments (**Figure 3B**).

### 3.5 Analysis of community structure composition and species differences among groups

**Figure 4:**
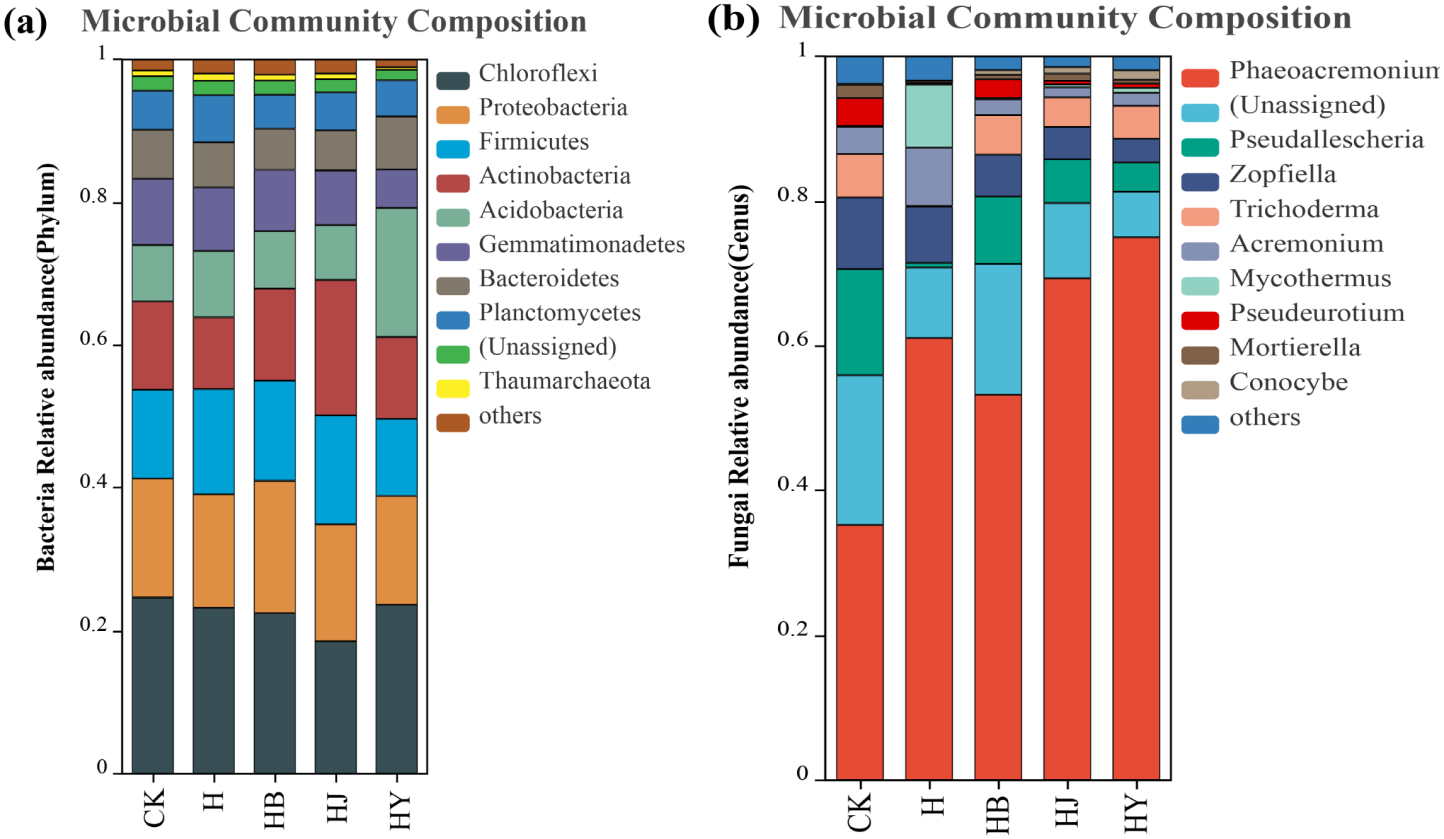
Effect of microalgal-based biofertilizer on the abundance of various **(A)** bacterial and **(B)** fungal species in the soil of potted tomato.

The community composition of the top 10 bacteria was analyzed at the phylum level, while the fungal community composition was analyzed at the genus level. The bacterial community was dominated by *Chloroflexi*, *Proteobacteria*, *Firmicutes*, *Acidobacteriota*, and *Actinobacteriota* (**Figure 4A**), followed by *Gemmatimonadates*, *Bacteroidota*, and *Planctomycetes*. Among the different treatment groups, the addition of microalgae-based biofertilizers to tomato soil with continuous cropping obstacles had varying effects on the relative abundance of dominant taxa. Specifically, only the *Proteobacteria* phylum showed a significant increase after the addition of HB, while the *Actinobacteriota* phylum increased significantly after the addition of HJ. The fungal community was dominated by genera such as *Phaeoacremonium*, *Pseudallescheria*, *Zopfiella*, *Trichoderma*, *Acremonium*, *Mycothermus*, and *Conocybe* (**Figure 4B**). The addition of microalgal biofertilizers significantly increased the abundance of the dominant fungal genus *Phaeoacremonium* and reduced the abundance of the pathogenic genus *Pseudallescheria*. Among them, the microalgae-based biofertilizer formed by HY had the most significant effect.

**Figure 5:**
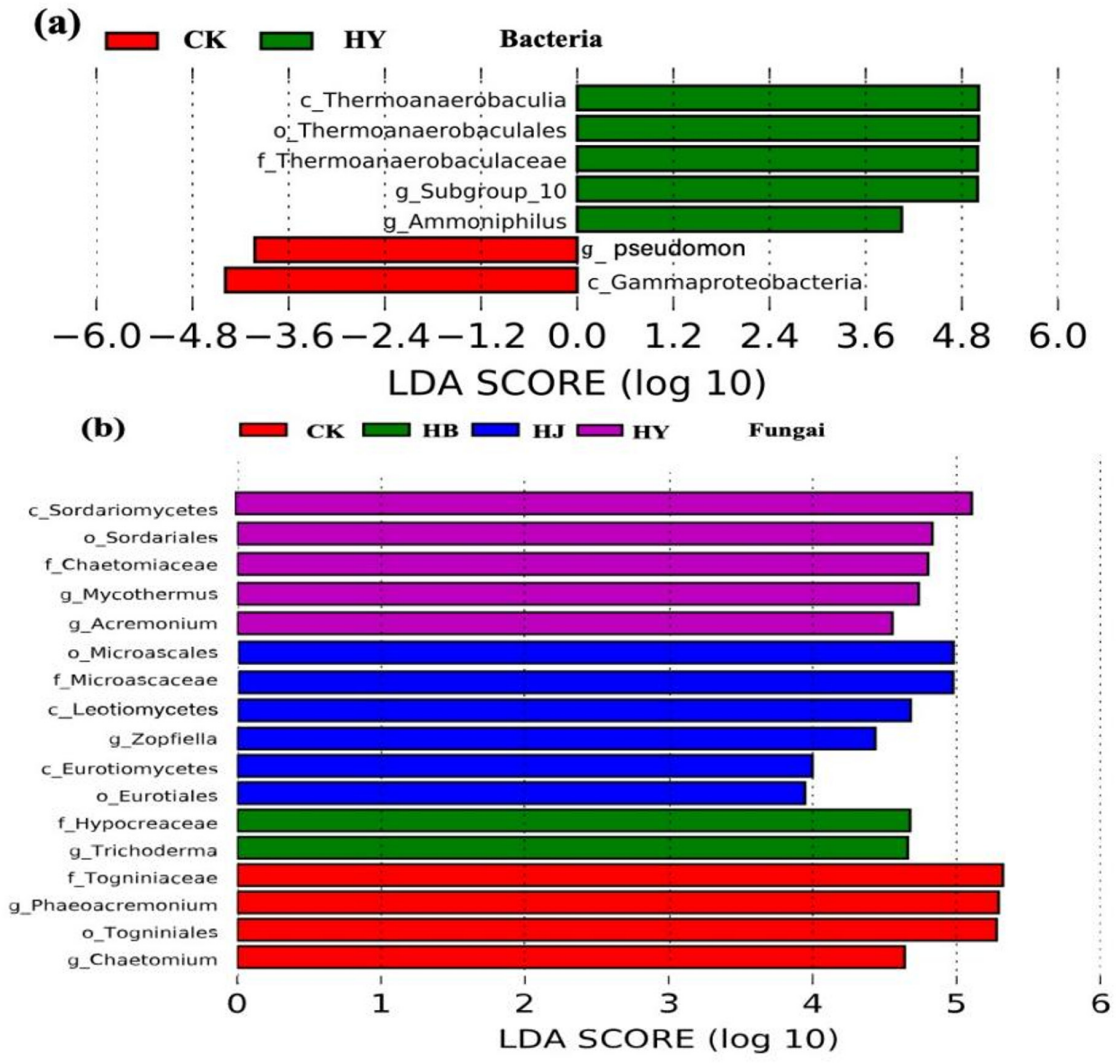
Line discriminant analysis (LDA) effect size (LEfSe) analysis of microbial abundance in the soil of potted tomato succession disorder. **(A, B)** Histogram of LDA scores computed for the differences in bacterial and fungal growth.

To further compare and analyze the differences within groups and identify species with significant differences in abundance among the groups, LEfSe was used to identify biomarker taxa at the genus level in tomato soil. The results showed that in the control group, pathogenic bacteria such as *Pseudomonas* were significantly enriched, while beneficial endophytes such as *Thermonaerobaculia*, *Subgroup_10*, and *Ammoniphilus* were significantly enriched in HY (**Figure 5A**). As for the fungal community, pathogenic fungi such as *Togniniaceae* and *Phaeoacremonium* were significantly enriched in the control group, while beneficial fungi such as *Sordariomycetes*, *Microascaceae*, and *Hypocreaceae* were significantly enriched in the HB, HJ, and HY groups (**Figure 5B**).

### 3.6 Correlation between microbial communities and physicochemical indices

**Figure 6:**
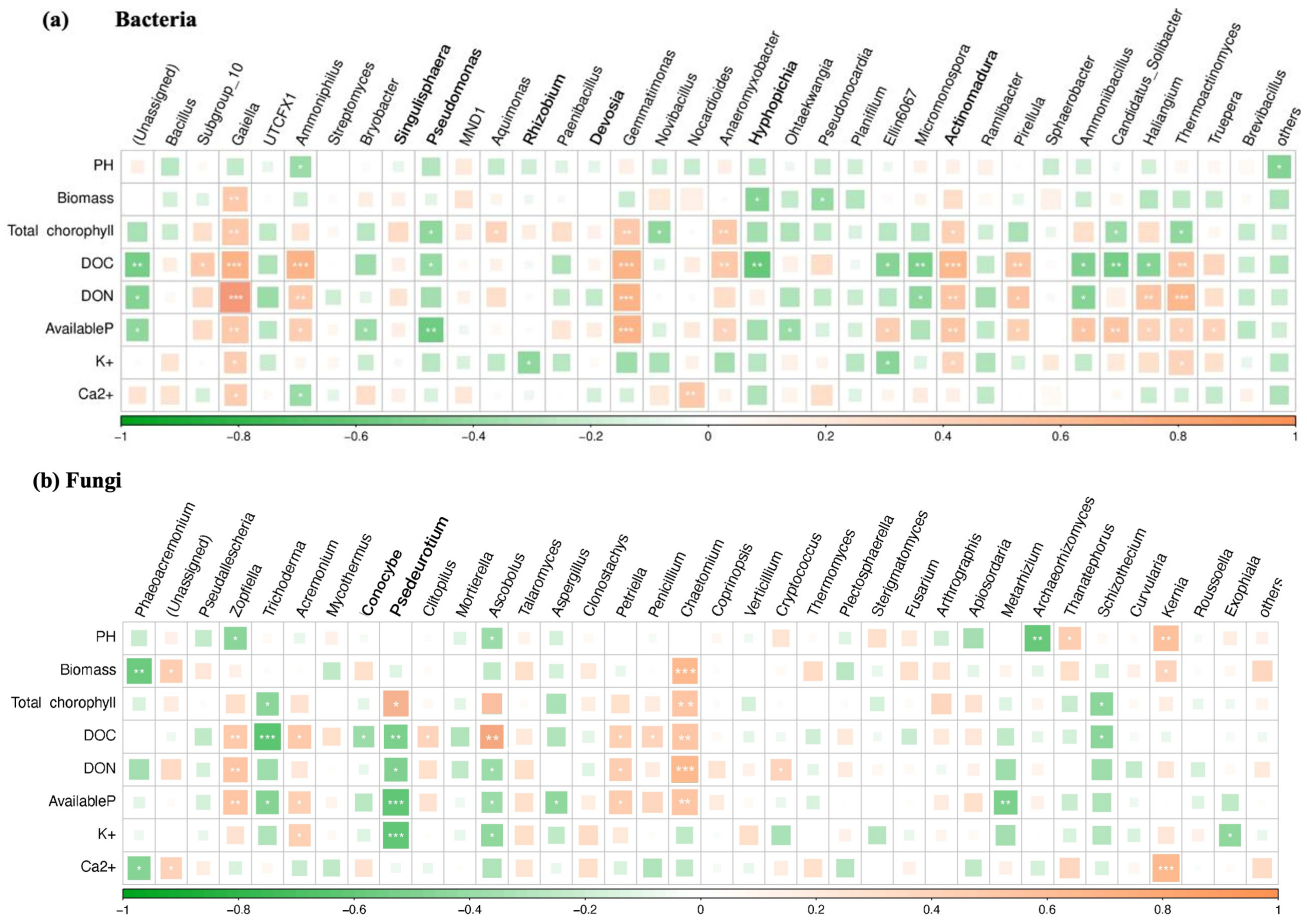
Correlation of the first 35 genera of **(A)** bacteria and **(B)** fungi with physicochemical properties in the soil of potted tomato. * represents p < 0.05, ** represents p < 0.01, and *** represents p < 0.001.

The Spearman rank correlation was used to investigate the relationship between environmental factors and microbial species richness (alpha diversity) at the genus level. Pairwise correlations and significant *P*-values were obtained. The results are shown in **Figure 6**. Among the bacteria, *Gaiella* and *Gemmatimonas* were significantly positively correlated with DOC and DON, *Ammoniphilus* and A*ctinomadura* were significantly positively correlated with DOC, *Thermoactinomyces* was significantly positively correlated with DON, AP was significantly positively correlated with *Gemmatimonas* and negatively correlated with *Pseudomonas*, and DOC was significantly negatively correlated with *Hyphopichia* (**Figure 6A**). As opposed to fungi, *Trichoderma* was significantly negatively correlated with DOC; *Psedeurotium* was significantly negatively correlated with AP and K^+^; *Zopfiella* and *Chaetomium* were significantly positively correlated with biomass, chlorophyll, and AP; and *Kernia* was significantly positively correlated with Ca^2+^ and pH (**Figure 6B**).

### 3.7 Functional cluster analysis of bacterial communities

FAPROTAX is a database of bacterial environmental functions. Based on the functional annotations and abundance information of the samples in the database, the top 15 most abundant functions were selected, and their abundance across each sample group was used to generate heat maps, which were clustered based on functional differences.

**Figure 7:**
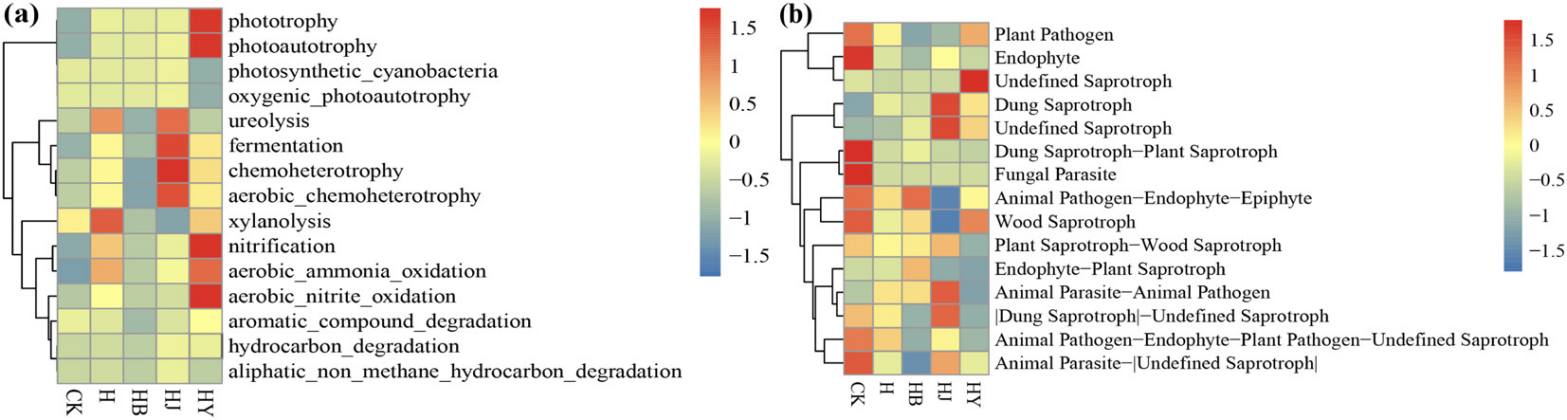
Heat map of **(A)** FAPROTAX and **(B)** FunGuild function annotation clustering.

HJ significantly increased chemoheterotrophy and HY significantly enriched nitrification, aerobic_ammonia_oxidation, and photoautotrophy compared to CK (**Figure 7A**), where the fermentation process plays an important role in the soil, promoting the degradation of organic matter, increasing soil fertility, and participating in the recycling of nutrients. Nitrification is a key step in the nitrogen cycle that helps bacteria convert ammonia and nitrogen into the nitrate form, which is more readily available for plant uptake. Aerobic_ammonia_oxidation converts ammonia, which is toxic to most organisms, into a less toxic form that can be utilized by plants and other organisms; the conversion of ammonia to nitrate also helps to provide nitrogen for plant growth. Therefore, microalgae-based fertilizer treatment induces a higher proportion of bacteria to participate in the soil carbon and nitrogen cycles in continuous cropping soils, which is conducive to the release of more inorganic nutrients from organic matter that are available to crops, increasing crop yields, improving soil functional degradation, and consequently alleviating crop succession barriers.

### 3.8 Functional relative abundance cluster analysis

FUNGuild is a database that categorizes the environmental functions of fungi, developed by classifying their ecological roles based on existing literature. This database facilitates the collection of ecological functions of fungal species found in the environment by utilizing species information obtained through amplicon analysis.

Based on the functional annotations and abundance information of the samples in the database, the top 35 functions and their formed abundances in each group of samples were selected, heat-mapped, and then clustered from the level of functional differences. Compared to CK, the application of HY reduced the abundance of inter-root plant pathogens, while the abundance of beneficial soil bacteria showed minimal change. However, the application of microalgae-based biofertilizer significantly decreased the abundance of inter-root plant pathogens in continuous tomato cultivation and notably increased the abundance of beneficial soil bacteria (**Figure 7B**). In summary, the algae-based biofertilizer effectively inhibits inter-root soil pathogens in continuous tomato cultures and promotes the growth of beneficial bacteria.

## 4 Discussion

### 4.1 Effect of microalgae-based biofertilizer on soil nutrients

As continuous cultivation progresses, essential nutrients required by crops become increasingly scarce in the soil, while unabsorbed elements accumulate, resulting in a nutrient imbalance (Zeeshan et al., 2023). Studies have shown that with the increase in cultivation duration, the soil TN and TP were decreased significantly. The excessive content of alkali-hydrolyzable nitrogen after continuous cotton cropping and the decrease in soil AP content indicate that continuous cotton cultivation disrupts the balance of N, P, and K in the soil (Ma et al., 2023). Carbon, nitrogen, and phosphorus are essential elements in microalgae cells. These elements participate in the synthesis of proteins, enzymes, chlorophyll, and genetic material, as well as energy production and metabolism, which play a crucial role in microalgae growth (Li et al., 2019; Singh et al., 2018). Microalgae can increase the content of available phosphorus in the soil and improve the microecological environment and fertility of the soil. DOC is composed of easily accessible carbon sources and can promote soil microbial metabolism, thereby competing with plant pathogens in the soil and inhibiting the development of crop diseases (Ramakrishnan et al., 2023). In this study, irrigating the soil of continuous tomato cropping with selected algae-based biofertilizers increased nutrient content to varying degrees. Among these, the HY treatment had the most significant impact, increasing AP, DOC, DON, NH4^+^-N, and NO_3_^−^-N levels by 27.4%, 231.3%, 403.4%, 125.2%, and 215.6%, respectively.

The results demonstrated that the combination of *Bacillus* sp. and microalgae significantly enhanced soil nutrient levels. This effect is likely due to the ability of *Bacillus* sp. to decompose organic waste in aquaculture environments into small molecules, such as carbon dioxide and nitrate, which improve water quality and mitigate the toxic effects of harmful substances on microalgae and soil microorganisms. In turn, the photosynthesis of microalgae provides oxygen for benthic aquatic animals and supports the decomposition of organic matter, creating a beneficial ecological cycle. This cycle enhances the diversity and functionality of microorganisms in both soil and plants. The combination of *Bacillus* sp. and microalgae likely facilitates the release and transformation of nutrients, making them more bioavailable for uptake by soil and plants. Additionally, the carbon sequestration and photosynthetic capacity of microalgae further optimize the chemical and biological properties of the soil, benefiting plant growth. By participating in the soil nutrient cycle, the microalgae-based biofertilizer increases the availability of essential elements for crop absorption, corrects nutrient imbalances, and enhances plant disease resistance in continuously cultivated soils.

Shell powder is rich in calcium, which is one of the nutrients required for microalgae and soil. The right amount of calcium can promote cell wall synthesis and growth for microalgae or microorganisms, making it more suitable for the soil environment. Straw fermentation liquid is rich in nitrogen, phosphorus, potassium, and other nutrients, which are also necessary for the growth of microalgae or microorganisms (Vona et al., 2024). The addition of shell powder and/or straw fermentation liquid can increase the organic matter content of the soil and improve the microbial community structure of the soil (Sharma et al., 2020). These microalgae-based biofertilizers are rich in nutrients that can be more easily absorbed and utilized by the soil, which would provide ample nutrients for the growth of plants.

### 4.2 Effect of microalgae-based biofertilizer on soil salinity

Continuous cultivation obstacles are also closely related to soil electrical conductivity and soluble salt ion content. Electrical conductivity is an important indicator for measuring the dissolved salt content in solutions (Zhang et al., 2024). EC measurement can reflect the migration and reactivity of salt ions in the solution. HB can reduce EC by 33.7% and is superior to employing *Tribonema* sp. alone, indicating that with the growth and absorption of *Tribonema* sp., some salt ions in the soil are utilized, providing a basis for reducing soil salinization. Compared to CK, HB can significantly increase the Ca^2+^ content in the soil by approximately 59.4%. The main component of shell powder is CaCO_3_, which is rich in Ca^2+^ itself. Studies have shown that trace elements such as K^+^, Ca^2+^, and Mg^2+^ are suitable and valuable for the metabolism and growth of microalgae (Matos et al., 2024), which is also consistent with the previous screening results of algae-based biofertilizer. The exchangeable magnesium content in the soil of greenhouse tomatoes with magnesium deficiency did not decrease. However, the composition ratio of the soil solid-phase exchangeable ions K^+^, Ca^2+^, and Mg^2+^ changed significantly, affecting the stability of the soil structure (Fei et al., 2024). HY has a better effect on Mg^2+^, which is likely due to the adsorption of Ca^2+^ and Mg^2+^ in soil by microalgae, thereby increasing their nutrient availability. Therefore, algae-based fertilizer can significantly improve soil nutrients, reduce salinity, ensure increased tomato yield and efficiency, and achieve efficient utilization of microbial biofertilizer and improvement of soil quality.

### 4.3 Effect of microalgae-based biofertilizer on microorganisms in tomato continuous cropping soils

Although there are many causes of crop failure, the most fundamental cause is the imbalance of soil microbiota and diversity, the reduction of beneficial microorganisms, and the enrichment of pathogenic microorganisms. These problems can lead to a variety of soil-borne diseases of plants (Yuan et al., 2022). Interactions between microalgae and pathogenic microorganisms may be affected by their metabolites (De Morais et al., 2015). However, there are currently limited studies on the use of microalgae combined with *Bacillus* sp. from agricultural and forestry waste to improve tomato continuous cropping obstacles and enhance soil fertility. In this article, compared with CK, the addition of three treatments of *Tribonema* sp. can significantly enrich beneficial endophytic bacteria in tomato continuous soil cultivation, such as *Bacillus*, *Rhizobium*, *Thermonaerobaculia*, *Subgroup_10*, *Ammoniphilus*, *Sordariomycetes*, *Microascaceae*, and *Hypocreaceae*, while reducing the abundance of harmful bacteria such as *Pseudomonas*, *Togniniaceae*, and *Phaeoacremonium*. *Sordariomycetes* is a reservoir of cellulases and active metabolites with great application potential, while *Microascales* can be used as a biocontrol agent in soil and water to prevent pests and pathogenic microorganisms. Additionally, there is a significant positive correlation between beneficial endophytic bacteria such as *Gaiella*, *Gemmatimonas*, *Ammoniphilus*, and A*ctinomadura* and DOC, DON, and AP. These findings demonstrate that the microalgae-based biofertilizer, created by combining *Tribonema* sp. with three types of agricultural waste, can alter the community structure of endophytic bacteria in tomato continuous cropping soil, promoting the growth of beneficial bacteria while inhibiting harmful bacteria. These resulting benefits can, therefore, reduce the occurrence of various soil-borne diseases.

Continuous cropping changes in soil microbial biomass, diversity, and microbiota have a major impact on the health of the soil, which in turn affects the growth of crops (Wani et al., 2015). Continuous cropping causes selective enrichment of microorganisms in the soil, an overall decline in bacterial populations, a significant rise in fungal populations, a dramatic increase in the number of pathogenic bacteria, and a shift in soil microorganisms from bacterial dominance to fungal dominance (Liu et al., 2020). In terms of richness and the Shannon index, the application of algae-based biofertilizer increased bacterial abundance while reducing the abundance of fungal pathogens more than tomato soil under 7 years of continuous cultivation. The HY group effectively inhibited pathogenic bacteria in the inter-root soil of continuously cultivated tomatoes and increased the abundance of beneficial bacteria, providing a theoretical basis for the positive impact of *Tribonema* sp. biofertilizer on plant growth.

The combination of microalgae and agricultural waste in algae-based biofertilizers provides an environmentally friendly and cost-effective alternative to traditional fertilizers. Microalgae-based biofertilizers alter soil fertility through the metabolism of their own active substances, thereby affecting the structure and function of soil microbial communities. However, the practical application of algal biofertilizers faces limitations, such as timeliness and scalability, which are key areas for future development. This study was limited to laboratory research, without large-scale application, and factors such as cultivation costs and insights from other literature must be considered. Additionally, the feasibility and effectiveness of fully or partially replacing chemical fertilizers with the current microalgae-based fertilizer warrant further investigation. Notably, the microalgae-based biofertilizers in this study make full use of agricultural by-products, promoting resource efficiency and creating a promising model for circular economy and sustainable agricultural practices.

## 5 Conclusions

Microalgae-based biofertilizer, as a biostimulant, can effectively improve the physicochemical properties and stress tolerance in the soil of potted tomatoes, which would be beneficial to the growth and development of the tomato crop. Compared with CK, the addition of all three microalgae-based biofertilizers was able to significantly enrich the soil of potted tomatoes with beneficial endophytes. Simultaneously, harmful bacteria decreased in abundance, and the beneficial endophyte groups *Gaiella*, *Gemmatimonas*, and *Ammoniphilus* were significantly and positively correlated with AP, DOC, and DON. These findings provide preliminary evidence that the application of microalgae-based biofertilizer can change the community structure of endophytic bacteria in the soil of potted tomatoes. This, in turn, promoted the growth and reproduction of beneficial bacteria and inhibited the growth of harmful bacteria. In addition, the novel fertilizer formed by combining *Tribonema* sp. with typical agroforestry waste in this study opens up the multi-pathway resource utilization of agroforestry waste and provides a theoretical basis for finding green and efficient biological organic fertilizers.

## Data Availability

The original contributions presented in the study are publicly available. This data can be found here: https://www.ncbi.nlm.nih.gov/sra/PRJNA1196298.
